# An epidemiological snapshot of cannabis use and comorbid substance abuse, depression and anxiety in young Romanians

**DOI:** 10.1192/j.eurpsy.2023.1416

**Published:** 2023-07-19

**Authors:** T. C. Ionescu, S. Zaharia, M. Simionescu, C. Tudose

**Affiliations:** 1 Department of Neuroscience, University of Medicine and Pharmacy “Carol Davila” Bucharest; 2 Department 2, Alexandru Obregia Clinical Hospital of Psychiatry; 3 University of Medicine and Pharmacy “Carol Davila” Bucharest, Bucharest, Romania

## Abstract

**Introduction:**

Cannabis consumption among Romanian youth has seen a steady increase in the last couple of decades.

**Objectives:**

This work attempts to fill the void left by the relative dearth of in-depth research on the subject of cannabis misuse in Romania, which is particularly concerning given the significant connection that exists between anxiety, depression, and cannabis usage.

**Methods:**

An epidemiological overview of cannabis misuse, mental comorbidities, and other socio-demographic characteristics was outlined through the use of validated self-reported scales on a small sample size (N=125) that was analyzed throughout this research. The purpose of this research was to outline this overview.

**Results:**

By applying the Cannabis Use Disorder Identification Test – Revised (CUDIT-R), Alcohol Use Disorder Identification Scale (AUDIT), Fagerstrom Test for Nicotine Dependence (FTND) and Hospital Anxiety and Depression Scale (HADS), the study’s results are as follows: although 48% of participants have tried cannabis, about a third (32%) have used it in the last 6 months. Among this sub-group, 40% presented scores that suggest Cannabis Use Disorder according to the DSM-V definition. Almost half (47%) percent of cannabis users had clinically significant scores for depression and anxiety, as opposed to 21% of non-users. Consumption of cannabis was more likely to be associated with alcohol abuse (63%) and nicotine dependence (85%). While students were equally represented among users and non-users; male gender and the unemployed were overly-represented. Interestingly, there was no correlation between relationship status and cannabis consumption.

**Conclusions:**

In conclusion, this study’s results are in line with most epidemiological literature regarding cannabis and can serve as a starting point for deeper, more analytical investigations of cannabis use in Romania.

**Disclosure of Interest:**

None Declared

